# Single-incision laparoscopic bariatric surgery

**DOI:** 10.4103/0972-9941.72397

**Published:** 2011

**Authors:** Chih-Kun Huang

**Affiliations:** Bariatric & Metabolic International (B.M.I.) Surgery Center, E-Da Hospital, Kaohsiung, Taiwan, 824

**Keywords:** Single-incision laparoscopic surgery, single-incision transumbilical laparoscopic surgery, SILS, bariatric surgery

## Abstract

**BACKGROUND:**

Bariatric surgery has been established as the best option of treatment for morbid obesity. In recent years single-incision laparoscopic surgery (SILS) has emerged as another modality of carrying out the bariatric procedures. While SILS represents an advance, its application in morbid obesity at present is limited. In this article, we review the technique and results of SILS in bariatric surgery.

**METHODS:**

The PubMed database was searched and totally 11 series reporting SILS in bariatric surgery were identified and analyzed. The case reports were excluded. Since 2008, 114 morbidly obese patients receiving SILS bariatric surgeries were reported.

**RESULTS:**

The procedures performed included SILS gastric banding, sleeve gastrectomy and gastric bypass. No mortality was reported in the literatures. Sixteen patients (14.05%) needed an additional incision for a liver retractor, a trocar or for conversion. Only one complication of wound infection was reported in these series. All the surgeons reported that the patients were highly satisfied with the scar.

**CONCLUSION:**

Because of abundant visceral and subcutaneous fat and multiple comorbidities in morbid obesity, it is more challenging for surgeons to perform the procedures with SILS. It is clear that extensive development of new instruments and technical aspects of these procedures as well as randomized studies to compare them with traditional laparoscopy are essential before these procedures can be utilized in day-to-day clinical practice.

## INTRODUCTION

Single-incision laparoscopic surgery (SILS) was first described as early as 1992 by Pelosi *et al*.[1,2] performed single-puncture laparoscopic appendectomy and hysterectomy. Currently SILS is considered to be a bridging technique to natural orifice transluminal endoscopic surgery (NOTES). This new approach minimizes the scars and is considered minimally invasive. Since the introduction of NOTES in 2004, researchers have used it for various surgical interventions.[3–5] Even with the worldwide popularity of NOTES, the techniques and instruments used are still being developed. On the other hand, SILS can be performed using refinements of existing technology and surgeons can perform SILS even with traditional laparoscopic instruments. Applications of SILS have expanded rapidly and various procedures including bariatric surgery have been carried out with this technique. Initially, SILS was used in bariatric procedures such as adjustable gastric banding (AGB) and sleeve gastrectomy because these procedures require the extension of a trocar incision for the placement of a subcutaneous port or for extracting the resected gastric specimen.[6–8] The incision was mainly in the upper abdomen in the beginning in this single-incision transabdominal (SITA) laparoscopic approach. It was felt that the patients would have a better cosmetic outcome if the SILS could be performed via a transumbilical incision as the umbilicus can hide the surgical wound, leaving no visible abdominal scars. Single-incision transumbilical (SITU) laparoscopic procedures seem to attract more surgeons because of the higher satisfaction from patients. Recently surgeons started to perform SILS in more complex bariatric procedures such as gastric bypass and bilopancreatic diversion procedures that require gastrointestinal anastomosis.[9–10] Here we review the surgical technique and results of SITA and SITU bariatric surgery.

## MATERIALS AND METHODS

We searched the PubMed for single-incision bariatric surgeries. A total of 11 case series were identified reporting 114 patients undergoing single-incision bariatric procedures [Table 1]. The procedures included AGB, sleeve gastrectomy and gastric bypass. Two routes were adopted - SITA and SITU and the surgeons chose either a single port or multiple ports through a single-incision to perform the procedures. Preoperative mean BMI and operative time were recorded for the different procedures. Conversion or placement of additional trocars during the procedures was also recorded. Most surgeons chose the SITU / multiple trocar approach. One complication of wound infection was reported.

**Table 1:** Case series of single-incision laparoscopic surgery bariatric surgery reported in the literature

Procedures	Author	Case	Route	BMI (mean)	Surgical time (mean)	Conversion /adding trocar	Complication
Gastric Band	Teixeira *et al*.	22	Transumbilical / multiple trocars	42	84	1 (4.4%)	0
	Huang *et al*.	3	Transumbilical / multiple trocars	38.1	62	0	0
	Saber *et al*.	8	Transumbilical / multiple trocars	38.9	105	1 (12.5)	0
	Keidar *et al*.	10	Transabdominal / multiple trocars	40.9	60	10(100%) Nathanson liver retractor	0
	Tacchino *et al*.	3	Transumbilical / single port	40.6	101	0	0
Total/Mean		46		40.87	82.1	12/46 (46.15%)	0
Sleeve gastrectomy	Saber *et al*.	7	Transumbilical / multiple trocars	49.3	143	1 (14.3%)	0
	Huang *et al*.	6	Transumbilical / multiple trocars	38.9	65.3	0	0
	Saber *et al*.	6	Transumbilical and Transabdominal / single port	52.8	123	3/6 (50%)	0
	Gentileschi *et al*.	8	Transumbilical /single port	56.2	128	0	1/8 (12.5%) Wound infection
Total/Mean		27		49.81	116.8	4/27 (14.81%)	0
Gastric bypass	Huang *et al*.	25	Transabdominal / multiple trocars	41.98	99.8	0	0
	Tacchino *et al*.	16	Transumbilical / single port	43.5	<120	0	0
Total/Mean		41		42.57	107.6	0	0

### Liver retraction in SILS bariatric surgery

**Figure 1:**
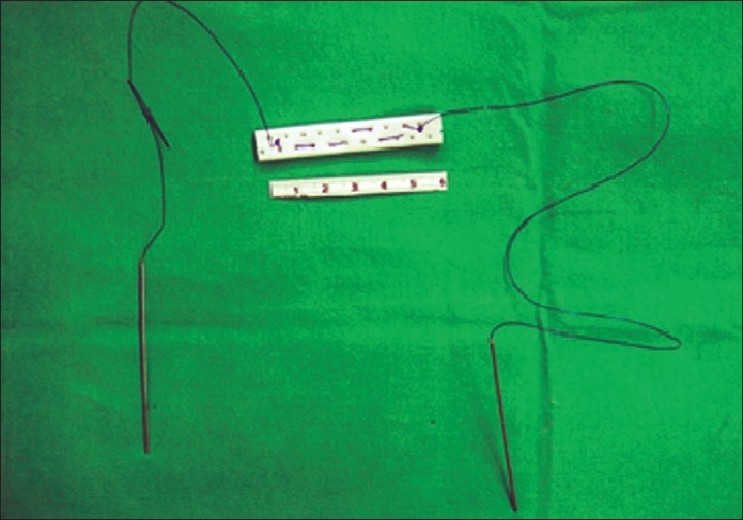
Design of liver suspension tape developed by Huang *et al*.

**Figure 2:**
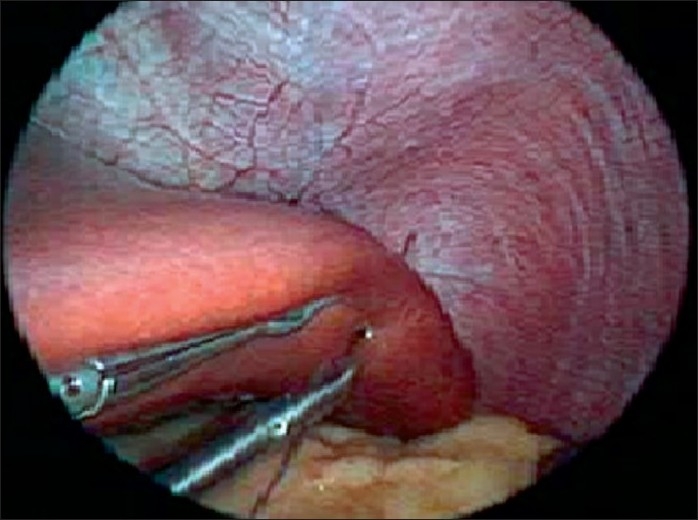
We measure a 6-cm length of a Jackson-Pratt drain, cut it and fix a with 2-0 polypropylene suture on either side.

**Figure 3:**
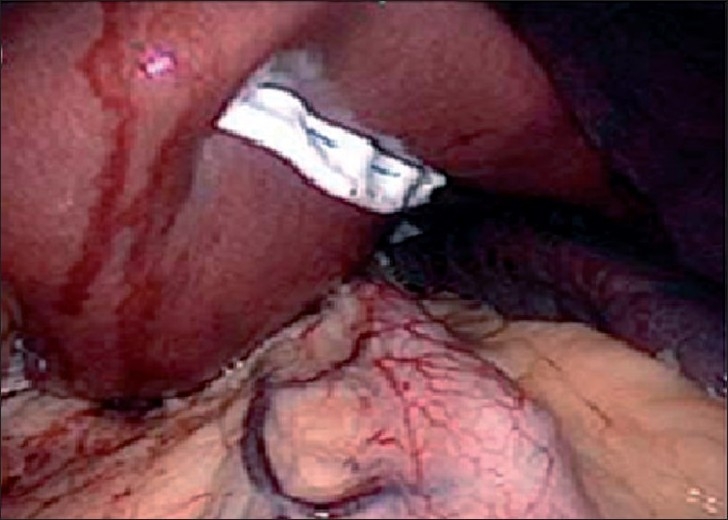
The lateral segment of the left liver is suspended by passing the suture through it.

For upper gastrointestinal laparoscopic surgery, liver retraction is necessary to ensure creation of an adequate working space. In morbidly obese patients, the hypertrophic left lobe of liver invariably hinders the surgeon’s view of the entire stomach. In multi-port bariatric procedures most surgeons use a Nathanson’s liver retractor via a subxiphoid incision to retract liver. To avoid the incision in SILS, retraction of the liver is a major challenge in SILS bariatric surgeries. Sakaguchi *et al*[11] invented a device for the retraction of the liver during conventional laparoscopic gastrectomy. However, in morbidly obese patients, the technique, which involves the dissection of the left triangular ligament of the left liver lobe, is more difficult to employ because most of these patients have a hypertrophic left liver lobe. Tacchino *et al*. used a transfixation suture, applied on the right crus and suspended outside as a liver retractor suture.[12,13] We have invented a new liver suspension tape technique that can be used to lift even massive livers in morbidly obese patients. [Figures 1–3].[9] Another non-puncturing method with a penrose drain and endow-hernia stapler has also been reported.[14] These techniques have been proved to be a quick and safe method in SILS bariatric surgery.

### SILS AGB

Laparoscopic AGB (LAGB) is considered to be the most physiological and safe bariatric surgery, not involving cutting and anastomosis of gastrointestinal tract.[15] Although the weight loss observed is slower, the procedure is popular. In AGB, the surgeons utilize the pars flaccida approach to place a band and then place 2-3 gastro-gastric sutures to hold it in place. It was believed that LAGB is a good surgery for bariatric surgeons to start the SILS bariatric procedure because it is a technically less demanding procedure and a 4-cm incision is required for placement of the port. Nguyen *et al*.[6] reported the first case of single-incision laparoscopic AGB. This is believed to be the first SILS bariatric surgery reported. Although the procedure was performed with a SITA method, it opened up the possibility of SILS in bariatric surgery. Keidar *et al*.[16] also used the SITA method by adding a liver retractor incision and the operative time was about 60 minutes. SITU-LAGB was reported by Teixeira *et al*.[17] and the patients included had neither hepatomegaly nor central obesity. Super-obese patients were also not considered for inclusion in this study. One conversion was observed in 22 reported patients. We also reported three cases with SITU method and Tacchino *et al*. used SILS port for the procedures.[12,18] But till now, there are no reported series comparing the outcomes of SILS LAGB and multiple-port LAGB.

### Sleeve gastrectomy

Sleeve gastrectomy is an emerging procedure for weight loss that provides rapid and satisfactory weight loss without any long-term vitamin deficiency. The procedure starts by mobilizing the greater curvature starting 6-cm from the pylorus till the angle of His. A vertical gastrectomy is then performed with endoscopic staplers. The resected stomach is extracted via an incision. The SILS approach was applied to sleeve gastrectomy as an incision was required for extraction of the resected gastric tube anyway. Saber *et al*. reported both SITU and SITA combined with a single port or multiple trocars.[19,20] They also compared the result of SILS and multi-port sleeve gastrectomy. Single-incision laparoscopic sleeve gastrectomy was associated with less postoperative pain, a lower need for analgesics and a decreased length of hospital stay compared to the conventional multi-port laparoscopic sleeve gastrectomy.[21] In these studies, most patients were superobese and one patient developed wound infection that required drainage.[22]

### Gastric bypass

**Figure 4:**
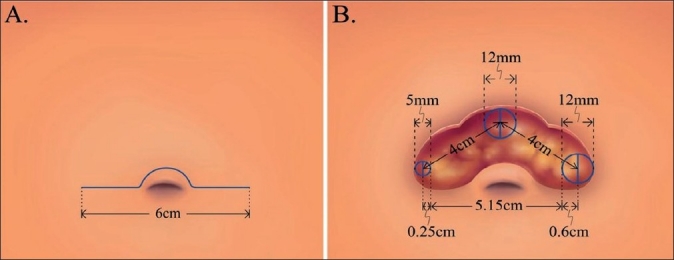
Design of single-incision transumbilical laparoscopic bariatric surgery (multiple ports) by Huang *et al*. Schematic of a 4-6-cm ω-incision in the suprumbilical area. (A) Schematic of the distance between the trocars (5 mm, 12 mm, 12 mm) that can reach (B) 4-cm more with this design thus increasing the space for manipulation.

**Figure 5:**
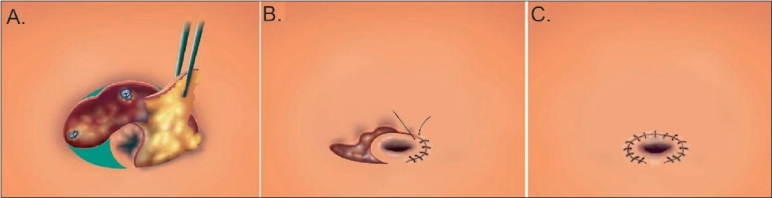
Closure of the ω-incision (A) At the conclusion of the surgery the trocars are removed and the fascia is repaired. The subcutaneous fat and skin at the angle is removed (Green area). (B) An umbilicoplasty is performed. (C) The wound becomes circular and is buried in the umbilicus.

Laparoscopic Roux-en-Y gastric bypass (LRYGB) has been considered as the gold standard of bariatric surgery. In the standard Roux-en-Y gastric bypass a 25-ml pouch is constructed and anastomosed to a Roux loop of jejunum. This is followed by closure of the mesenteric defect. Till now only two authors have reported the results of the SILS approach for gastric bypass. Tacchino *et al*. elongated the gastric pouch to 6 cm in length to speed up the dissection and decrease the tension on the gastrojejunal anastomosis. Two gastric bypass procedures were adopted - 16 patients receiving a single loop and two receiving a double loop. The single-loop gastric bypass involved only one anastomosis, and was thought to decrease the difficulties of technique.[23] We developed a novel method using a SITU approach and subsequently performing an ω-umbilicoplasty for the Roux-en-Y gastric bypass.[18,24] The increased space of manipulation in the 6-cm incision and subsequent umbilicoplsty design makes the procedure easier, saves time and is still scarless [Figures 4–5]. In fact, the procedures needs modifications including use of an Endostich device for sutures and some stay sutures for counter traction. No complications were observed in these two reports.

### Bilopancreatic diversion

Biliopancreatic diversion is a malabsorptive technique of bariatric surgery that has gained wide acceptance especially at superobesity. It is performed by carrying out a vertical sleeve gastrectomy combined with a duodenoenterostomy. The surgery is considered to be the most complex bariatric procedure. Tacchino *et al*. reported the first case with SITU / single-port method[10] in a 57-year-old man with a body mass index of 43 kg/m^2^. The procedure took 130 minutes to finish and there were no complications.

### Complications

In the 114 patients analysed here, only one complication of wound infection was reported in the SITU group. This was accomplished by carefully selecting the patients with minimum comorbidities in the learning stage. Another reason was that most of these surgeons were very experienced in the multi-port bariatric surgery. Placement of additional trocars whenever required is considered prudent in these challenging procedures to avoid intraoperative complications. The wound care after SILS procedures needs to be addressed. Abrasions and ecchymosis of the SILS wound due to manipulation of instruments and trocars may lead to a longer time for caring of the wound. Careful closure is mandatory to avoid the possibility of incisional hernia developing in the long term.

## DISCUSSION

SILS has recently gained acceptance in bariatric surgery as the procedure has possible benefits. It is an alternative to NOTES - an experimental procedure whose feasibility is frequently debated.[17,18] The surgical technique involved is almost identical to that required for conventional laparoscopic surgery. If the surgery is performed transumbilically, the surgical scar is almost completely hidden inside the belly button and the surgical site is scarless. Although some surgeons argue that very obese patients are not concerned about the scarring up to 70% of patients undergoing bariatric procedures are women and consider scarring to be an important factor. We have done a comparative study in SITU-LRYGB and 5-port LRYGB (unpublished data). The promising result showed better patient satisfaction regarding the cosmesis in the SITU group. Also, though the operation times were longer, the recovery and hospitalization was similar in both groups.

Patient selection is important for the single-incision bariatric surgery and some patients are not well-suited for these procedure. Most authors do not recommend this procedure for those with a BMI greater than 50 because of abundant abdominal fat that makes surgery very difficult, especially for a LRYGB, which needs anastomosis technique. Also, due to the longer-than-normal working distance between the angle of His and the umbilicus in the SILS procedure, it should be avoided in tall patients (height >180 cm). Despite its advantages in SILS bariatric surgery, the small umbilical incision tends to “crowd” the trocars in a very limited surgical field. The resulting reduced instrument triangulation and inability to retract tissue by the assistant make this procedure more arduous. It is essential to use a 30º 5-mm laparoscope to avoid conflict with other surgical instruments. Some surgeons have used a semi-flexible endoscopic camera system to make the procedure more comfortable. In addition, handling a hypertrophic liver and abundant visceral fat is also critical in morbidly obese patients. Longer endoscope, longer graspers and longer endocutter are also highly recommended. As this procedure requires far more skill than a conventional 5-port surgery it should only be undertaken by very experienced bariatric surgeons.

## CONCLUSIONS

In conclusion, SILS bariatric has been shown to be a technically feasible and reproducible procedure for a select group of morbidly obese patients. Because of the abundant visceral and subcutaneous fat and multiple comorbidities in morbid obesity, it is more challenging for surgeons to perform the procedures with SILS. It is clear that extensive development of new instruments and technology will make these procedures easier to perform. Nevertheless, randomized studies to compare the SILS bariatric procedures with traditional multi-port surgery are essential to further develop this highly technique-dependent surgery.
